# Impact of incentive and selection strength on green technology innovation in Moran process

**DOI:** 10.1371/journal.pone.0235516

**Published:** 2020-06-30

**Authors:** Runtian Zhang, Jinye Li

**Affiliations:** School of Economics and Management, Xinjiang University, Urumqi, China; Shandong University of Science and Technology, CHINA

## Abstract

Methods of previous researches on green technology innovation will have difficulty in finite population. One solution is the use of stochastic evolutionary game dynamic-Moran process. In this paper we study stochastic dynamic games about green technology innovation with a two-stage free riding problem. Results illustrate the incentive and selection strength play positive roles in promoting participant to be more useful to society, but with threshold effect: too slighted strength makes no effect due to the randomness of the evolution process in finite population. Two-stage free riding problem can be solved with the use of inequality incentives, however, higher inequality can make policy achieves faster but more unstable, so there would be an optimal range. In this paper we provided the key variables of green technology innovation incentive and principles for the environmental regulation policy making. Also reminded that it’s difficult to formulate policies reasonably and make them achieve the expected results.

## Introduction

Since the publication of 《game theory and economic behavior》 by Von Neumann & Morgenstern [1] in 1944, people have begun to analyze the conflict and competition in politics, economy and social according to the game method. How to promote cooperation and altruistic behavior has always been the focus of game theory research [2] [3].

But a lot of problems cann’t be explained under the assumption of rational man, even should not have occurred such as the tragedy of commons. In 1957, concept of bounded rationality was put forward by Simon [4]. Smith [5] developed evolutionary game theory in 1982 and proposed a practical tool for studying the dynamics of natural selection: evolutionary stable strategy, to represent the stable state of evolutionary game. In 1978, Taylor & Jonker [6] used replicator dynamic to represent the dynamic convergence process to a stable state. Evolutionary game theory is of great practical significance, which provides a great use for biology [7] and various social sciences especially economics [8].

Evolutionary game dynamics involves deterministic and stochastic evolutionary game dynamics [9] [10] [11] [12]. Deterministic evolutionary game dynamics studies the mixed infinite population, usually the participant's attribute influences and determines the game strategy and the successful strategy spreads in the group [13], which can be described by replicator dynamics equation [14] [15] [16].

Green Technology innovation refers to the process for enterprises to replace the original products with more environmentally friendly products by updating process and reforming equipment under the pressure of external system or market demand and other induced factors [17]. It promotes sustainable development by reducing pollutant emissions, improving fuel combustion efficiency and changing enterprise production mode [18] [19] [20] [21]. However, as the supplier of products, enterprises are lacking the enthusiasm and initiative of green technological innovation when facing with high risks of technological innovation, high investment in R&D and the uncertain factors in the market [22]. Moreover, the production activities of enterprises are often locked in the high carbon mode due to the effect of technological lock-in [23]. Reasonable environmental regulation policy is conducive to innovation compensation effect [24] to stimulate the innovation behavior of enterprises, the theory has been proved to be effective in empirical researches [25]: Testa [26] found that environmental protection standards will stimulate technology research and development of enterprises, Rassier [27] found that regulatory system can promote new technology R&D of enterprises and have a positive impact on business performance.

In recent years, many scholars have studied the relationship between environmental regulation and enterprise technological innovation using evolutionary game method, mainly involving economic incentives and institutional designs [28] [29] [30]: Estalaki [31] used the heuristic game optimization method to study the river water quality management. The analysis shows that the reward and punishment system has a certain impact on the quality of water. Huang [32] studied the supply chain model under the government subsidy mechanism and the centralized control mechanism without subsidy based on the duopoly environment and found that compared with the centralized control, incentive is more effective in promoting technological innovation and protecting the environment. Wang [33] set up a dynamic game model of government and enterprise to study the impact of different government subsidies on green technology innovation decision-making in different stages of innovation. Krass [34] built a Stackelberg game model between the government and enterprises and found that the combination of environmental taxes and subsidies can encourage enterprises to adopt low-carbon emission reduction technology. Enterprises will choose different green innovation modes mainly affected by the government green innovation subsidies, carbon tax and other environmental policies [35]. However, single environmental regulation method cannot stimulate more green innovation, only a variety of methods can produce better results [36].

Existing studies in this field have mainly focused on replication dynamic equation. Though it has good mathematical characteristics, it can only describe the deterministic evolutionary dynamics in infinite population [37] [38]. The randomness in finite population plays an important role so it will bring noise interference if we continue to use the replicator dynamics equation, and the long-term stable state will cannot be captured. The result in infinite population cannot be simply recursive in finite population, therefore stochastic evolution dynamic game model which it is more realistic to explore the situation in finite population is developed [39] [40] [41] [42] [43]. The evolutionary game dynamics in finite population is described as a stochastic process based on the assumption of bounded rationality and insufficient information, the stochastic process method is used to analyze the process of reaching equilibrium [44].

Previous studies of stochastic evolutionary game in finite population mainly focused on the renewal mechanism of research strategy: synchronous update process and asynchronous update process [45]. The former one which mainly including Moran process and Fermi process refers to that a participant is selected to produce a replica according to its fitness and randomly replace a participant in the population at each time step, so that the population remains unchanged [46] [47]. The latter one which mainly including Wright-Fisher process refers that all participants produce replicas at the same time and then select the next generation from the replicas, so that the population remains unchanged [48] [49].

For the situation of this paper, each participant can make decision at any time independently, so synchronous update process is more likely to be used. Further, replacement possibilities in Fermi process are obtained by comparing participants in pairs [50], whose in Moran's are by comparing to the other *N* − 1 participants [51]. Moran process is more in line with the requirements in fully mixed finite population in this paper.

Moran process describes the dynamic process following Markov state transition matrix [52]. Taylor showed up the difference between the evolution in Moran process and the replicator dynamics equation in infinite population. Nowak [53] gave the expression of the expected return of all kinds of participants and deduce the changes of replacement probabilities before and after the process. Traulsen [54] explored the functions of the expected return under strong and weak strength of selection. Moran process is not only widely used in sociology and theoretical biology [55], also in behavioral economics: Chai [56] studied the evolution of gross income maximization strategy and net profit maximization strategy in the process of manufacturing strategy selection. Wang [57] applied Moran process to the evolution of consumer crowdfunding strategy selection.

To address the existing knowledge gaps in the evolutionary game of green technology innovation, two aims are mentioned while using a number of different parameters and combinations in Moran process model:

(1) Summarize the dynamic nature of the relationship between benefits of different strategies and the probabilities of selecting them. (2) Determine how the strength of natural selection, the strength of incentives and differential coefficient are related to variations in participants strategies over time. Those highlight how the importance of properly designed policies, that is, how the benefits of participants should be consistent with the input, rather than promoting free riding strategies.

In Chapter 2 we introduce the basic settings and quantify the benefits of different participants in each period. This is followed by the Moran process method, simulations and key conclusions with results in Chapter 3.1, 3.2 and Chapter 4, respectively. The theoretical and practical implications, implications and the future research directions are involved in Chapter 5.

## Model

In this study, we establish a two-stage stochastic evolutionary game model in finite population, each participant can choose strategy “innovate” or strategy “do not innovate” in the first one, when all participants choose innovate, strategy “leading” or strategy “following” will be chosen in the second stage.

### The first stage (*t*_0_-*t*_1_)

We assume the green technology innovation reward takes path of the marginal cost reduction in producing process. The marginal cost for a non-innovator is *C*_1_(1<*C*_1_) and for innovators reduces to 1. The innovators have absolute dominance and form a monopoly during this period. By setting the price to *C*_1_, the innovating reward is

$$R_{1}=(C_{1}-1)\frac{aR}{C_{1}}$$ (1)

The term *R* is used here to refer to all the reward through green technology for the society, and term *a* (0 < *a* < 1) is the proportion of innovators’ share of the reward *R*.

Ecological damage can be regarded as public goods with negative externalities, so if there is no innovator, each participant bears a loss −R* because no one promotes green progress [58], *R* = *N* × R* (N is the population size).

### The second stage (*t*_1_-*t*_2_)

Analogy with the assumption of Chapter2.1, the marginal cost for both leading innovators and following innovators is 1, the price is still set to *C*_1_. Reward *R* is divided equally between the leading innovator and the following innovator

$$R_{1}^{*}=R_{2}=(C_{1}-1)\frac{bR}{2C_{1}}$$ (2)

Where *b* is the proportion of two kinds of innovators’ total share of the reward *R* after the market changed from absolute monopoly to duopoly. Since the intervention of following innovators has a crowding-out effect on the profits of the leading innovators, 0 < *ab* < 2*a*.

The total discounted utility of the leading innovators used the expression of discount utility of technology patents defined by An [59], it is assumed *t*_0_ = 0 and all types of selectors are risk-neutral, the preference function is linear and the coefficient is 1.

$$V_{1}=\int_{t_{0}}^{t_{1}}e^{-rt}R_{1}dt+\int_{t1}^{t_{2}}e^{-rt}R_{1}^{*}dt-=\frac{R_{1}}{r}(1-e^{-rt_{1}})+\frac{R_{1}^{*}}{r}(e^{-rt_{1}}-e^{-rt_{2}})$$ (3)

Where r is the discount rate of future utility. And the total discounted utility for following innovators:

$$V_{2}=\frac{R_{2}}{r}(e^{-rt_{1}}-e^{-rt_{2}})$$ (4)

## Computational method and simulation

### The Moran process

In order to inspire more participants to choose “innovate” strategy, an incentive reward which can be gotten after innovate successfully for participant is considered in this model.

We consider 2 × 2 strategies in finite population *N*. The payoff matrix of strategies symmetric game between “innovate(V)” and “do not innovate(D)” given by

Where *C* is the input of R&D on green technology innovation, *P*(*C*) is the probability to succeed (P′^(*C*)^ > 0, P″^(*C*)^ < 0), *T*(0 < *T* < 1) is the strength of incentives, so the net reward is *P*(*C*)*T* − *C*.

According to the Table 1. when the number of “innovate” participants is *i* and of “do not innovate” participants is *N* − i in a fully mixed population sized *N*, the expected returns of “V” participants and “D” participants are

$$U_{V}^{i}=\frac{i-1}{N-1}\left[P\left(C\right)\frac{(C_{1}-1)(1-e^{-rt_{2}})bR}{NrC_{1}}-C+P\left(C\right)T\right]+\frac{N-i}{N-1}\left[P\left(C\right)\frac{(C_{1}-1)(1-e^{-rt_{2}})aR}{NrC_{1}}-C+P\left(C\right)T\right]$$ (5)

i=1,2,3,…,N−1

$$U_{D}^{i}=\frac{N-i-1}{N-1}(-R^{*})$$ (6)

i=1,2,3,…,N−1

**Table 1:** Payoff matrix of “V” and “D”.

	Innovative(V)	Do not innovative(D)
Innovative(V)	$P\left(C\right)\frac{(C_{1}-1)(1-e^{-rt_{2}})bR}{NrC_{1}}-C+P\left(C\right)T$	$P\left(C\right)\frac{(C_{1}-1)(1-e^{-rt_{2}})aR}{NrC_{1}}-C+P\left(C\right)T$
Do not innovative(D)	0	−R*

In evolutionary game algorithm, the fitness of the strategy depends on the expected return of the strategy, the diffusion rate of the strategy is positively related to the expected return of the corresponding strategy. At present, there are two ways to express the fitness: linear mapping and exponential mapping. Although exponential mapping is more advantageous under strong selection intensity [60], more evidence shows that participants make decisions under weak selection intensity [61] [62]. Moreover, frequency-dependent Moran processes [63] has convenient properties for the analysis of weak selection, different mapping forms of fitness will not change the qualitative results, but only the diffusion speed of process, so linear mapping is still worked and widely used [64].

Therefore, we choose linear mapping form, the fitness of strategy V and strategy D is the linear function of their expected return [65]

$$f_{V}=1-u+uU_{V}^{i}\;,f_{D}=1-u+uU_{D}^{i}$$ (7)

Where *u* ∈ (0,1) is the strength of natural selection, which indicates the sensitivity of the participant to the payoffs with different strategies.

At every time step it will take fitness as probability to generate a new participant to randomly replace an old selector. As we pointed above, the probability of the new participant choosing strategy “V” is $\frac{if_{V}}{if_{V}+(N-i)f_{D}}$, and the probability of the new participant choosing strategy “D” is $\frac{(N-i)f_{D}}{if_{V}+(N-i)f_{D}}$, and there is 1% mutation rate.

So at every time step in the frequency-dependent Moran process, the number of “innovate” participants maybe adds 1/reduces 1/holds, but the population size is always *N*. Therfore, the Markov probability transfer matrix of Moran process is a tridiagonal matrix, the diagonal element is

$$P_{i,i+1}=\frac{if_{V}}{if_{V}+\left(N-i\right)f_{D}}\times \frac{N-i}{N}$$ (8)

$$P_{i,i-1}=\frac{\left(N-i\right)f_{D}}{if_{V}+\left(N-i\right)f_{D}}\times \frac{i}{N}$$ (9)

$$P_{i,i}=1-P_{i,i+1}-P_{i,i+1}$$ (10)

and the other elements are 0.

Moran process has two stable states: *i* = 0, and *i* = *N*, which means all participants will all choose strategy “V” or strategy “D”. Hence we can get from the total probability formula

$$\{\begin{array}{ll} F_{0,V}=0 & \\ F_{i,V}=P_{i,i+1}F_{i+1,V}+P_{i,i-1}F_{i-1,V}+P_{i,i}F_{i,V} & i=1,2,3,\ldots ,N-1\\ F_{N,V}=1 & \end{array}$$ (11)

Where *F*_*i*,*V*_ is the distribution function from the initial state *i* to all *N* participants choose strategy “innovate V”.

$$q_{i}=F_{i,V}-F_{i-1,V}$$ (12)

$$\sum_{i=1}^{N}q_{i}=1$$ (13)

$$q_{1}=\frac{1}{1+\sum_{i=1}^{N-1}\prod_{k=1}^{i}\frac{P_{i,i-1}}{P_{i,i+1}}}$$ (14)

$$q_{i}=q_{1}(1+\sum_{i=1}^{N-1}\prod_{k=1}^{i}\frac{P_{i,i-1}}{P_{i,i+1}})=\frac{1+\sum_{i=1}^{i-1}\prod_{k=1}^{i}\frac{f_{D}}{f_{V}}}{1+\sum_{i=1}^{N-1}\prod_{k=1}^{i}\frac{f_{D}}{f_{V}}}$$ (15)

If the initial state is *i* = 1, the replacement probability of strategy “V” will be

$$Q_{V}=q_{1}=\frac{1}{1+\sum_{i=1}^{N-1}\prod_{k=1}^{i}\frac{f_{D}}{f_{V}}}=\frac{1}{1+\sum_{i=1}^{N-1}\prod_{k=1}^{i}\frac{1-u+uU_{D}^{i}}{1-u+uU_{V}^{i}}}$$ (16)

If the initial state is *i* = *N* − 1, the replacement probability of strategy “D” will be

$$Q_{D}=1-q_{N-1}=\frac{1}{1+\sum_{i=1}^{N-1}\prod_{k=1}^{i}\frac{f_{V}}{f_{D}}}=\frac{1}{1+\sum_{i=1}^{N-1}\prod_{k=1}^{i}\frac{1-u+uU_{V}^{i}}{1-u+uU_{D}^{i}}}$$ (17)

The ratio of fixed point probability of strategy “V” and strategy “D”

$$\frac{Q_{V}}{Q_{D}}=\frac{1+\sum_{i=1}^{N-1}\prod_{k=1}^{i}\frac{1-u+uU_{V}^{i}}{1-u+uU_{D}^{i}}}{1+\sum_{i=1}^{N-1}\prod_{k=1}^{i}\frac{1-u+uU_{D}^{i}}{1-u+uU_{V}^{i}}}=\prod_{i=1}^{N-1}\frac{1+u(U_{V}^{i}-1)}{1+u(U_{D}^{i}-1)}$$ (18)

$\frac{Q_{V}}{Q_{D}}>1$ means strategy “V” has more invasion dynamic to invade strategy "D”, strategy “V” is more likely to defeat strategy “D” and become evolutionary stability strategy. As a result, The disadvantaged position of strategy "D” in the evolution process will eventually make them completely replaced by strategy "V”, all the participants will become innovators.

Moreover, what is more realistic is that nonobjective factors such as decision makers' emotions, preferences, social responsibility affect their decision-making, which is not entirely based on expected return, so in this evolutionary game model, we consider the evolution trend of strategy choice under weak selection(*u* → 0). Taylor expansion of the solution above at *u* → 0

$$\frac{Q_{V}}{Q_{D}}=\prod_{i=1}^{N-1}\frac{1+u(U_{V}^{i}-1)}{1+u(U_{D}^{i}-1)}\approx \prod_{i=1}^{N-1}[1+\left(U_{V}^{i}-U_{D}^{i}\right)u+\delta ]$$ (19)

**Conclusion 1**: strategy “V” invades with strength of natural selection increases.

$$\begin{array}{l} \frac{Q_{V}}{Q_{D}}\approx \prod_{i=1}^{N-1}1+\{\frac{i-1}{N-1}\left[P\left(C\right)\frac{(C_{1}-1)(1-e^{-rt_{2}})bR}{NrC_{1}}-C+P\left(C\right)T\right]+\frac{N-i}{N-1}\left[P\left(C\right)\frac{(C_{1}-1)(1-e^{-rt_{2}})aR}{NrC_{1}}-C+P\left(C\right)T\right]\\ \;-\frac{N-i-1}{N-1}(-R^{*})\}u+\delta \end{array}$$ (20)

Extract the common factor containing *T*

$$\frac{Q_{V}}{Q_{D}}\approx \prod_{i=1}^{N-1}\left[1+P\left(C\right)Tu+\delta +\eta \right]$$ (21)

**Conclusion 2**: strategy “V” invades with the strength of incentives increases.

Further more, strategy “V” becomes the evolutionary stability strategy, all the participants choose to innovate, there is still a problem called second-order free riding problem in the second stage: Following innovators are not willing to pay R&D costs but still want to take benefits by following to leading innovators with little cost. The central argument of free riding theory is that once public good exists, every member of society can enjoy the benefits whether they have contributed to it or not. This characteristic of public goods determines that everyone in a group of rational people may want others to work hard to achieve the goal, while he or she will enjoy it.

But if all the participants want to be the following innovators, they will all become non-innovators as a result. There is additional incentive method needed. Olson [66] put forward a series of ways to solve the free riding dilemma. The basic idea is that though public goods provide a collective incentive, it’s not enough for a rational person to strive for certain public goods, selective incentive is necessary. Selective incentive means that you will lose or be not qualified to get something if don't participate in an action. There are several kinds of selective incentive, which of them most workable in this situation is "principle of inequality" [67]. If an individual or a small group can get more rewarded from making a direct contribution independently, then may contribute to a certain cause alone. We set the differential coefficient *μ* ∈ [1,2]: the incentives giving to leading innovators are *T*_1_ = *μT*, and the incentives giving to following innovators are *T*_2_ = (2 − *μ*)*T*, thus $\frac{T_{1+T_{2}}}{2}=T$.

Still consider a 2 × 2 strategies in size *N*. The payoff matrix of strategies symmetric game between “innovate(L)” and “do not innovate(F)” given by

However, followers may misreport that he/she is a leading innovator when asymmetric information exists, so the cost and accuracy of judging whether the information is true or false will also seriously affect the final results. It should be valued in practice though this problem is not in the scope of this article.

According to the Table 2. when the number of “leading” participants is *i* and of “following” participants is *N* − *i*, the expected returns of “L” participants and “F” participants are

$$U_{L}^{i}=\frac{i-1}{N-1}\left[P\left(C\right)\frac{(C_{1}-1)(1-e^{-rt_{2}})bR}{NrC_{1}}-C+\mu P\left(C\right)T\right]+\frac{N-i}{N-1}\left[P\left(C\right)\frac{(C_{1}-1)(1-e^{-rt_{1}})aR}{NrC_{1}}+P\left(C\right)\frac{(C_{1}-1)(e^{-rt_{1}}-e^{-rt_{2}})bR}{NrC_{1}}-C+\mu P\left(C\right)T\right]$$ (22)

i=1,2,3,…,N−1

$$U_{F}^{i}=\frac{i}{N-1}\left[P\left(C\right)\frac{(C_{1}-1)(e^{-rt_{1}}-e^{-rt_{2}})bR}{NrC_{1}}+\left(2-\mu \right)P\left(C\right)T\right]+\frac{N-i-1}{N-1}(-R^{*})$$ (23)

i=1,2,3,…,N−1

**Table 2:** Payoff matrix of “L” and “F”.

	Leading innovation	Following innovation
Leading innovation	$P\left(C\right)\frac{(C_{1}-1)(1-e^{-rt_{2}})bR}{NrC_{1}}-C+\mu P\left(C\right)T$	$P\left(C\right)\frac{(C_{1}-1)(1-e^{-rt_{1}})aR}{NrC_{1}}+P\left(C\right)\frac{(C_{1}-1)(e^{-rt_{1}}-e^{-rt_{2}})bR}{NrC_{1}}-C+\mu P\left(C\right)T$
Following innovation	$P\left(C\right)\frac{(C_{1}-1)(e^{-rt_{1}}-e^{-rt_{2}})bR}{NrC_{1}}+\left(2-\mu \right)P\left(C\right)T$	−R*

After calculations which are similar to(7)–(17), the ratio of fixed point probability of strategy “L” and strategy “F”

$$\frac{Q_{L}}{Q_{F}}=\frac{1+\sum_{i=1}^{N-1}\prod_{k=1}^{i}\frac{1-u+uU_{L}^{i}}{1-u+uU_{F}^{i}}}{1+\sum_{i=1}^{N-1}\prod_{k=1}^{i}\frac{1-u+uU_{F}^{i}}{1-u+uU_{L}^{i}}}=\prod_{i=1}^{N-1}\frac{1+u(U_{L}^{i}-1)}{1+u(U_{F}^{i}-1)}$$ (24)

$\frac{Q_{L}}{Q_{F}}>1$ means strategy “L” has more invasion dynamic to invade strategy "F”, strategy “L” is more likely to defeat strategy “F” and become evolutionary stability strategy. As a result, the disadvantaged position of strategy "F” in the evolution process will eventually make them completely replaced by strategy "L”, all the participants will become leading innovators.

As we pointed above, what is more realistic is under weak selection(*u* → 0). Taylor expansion of the solution above at *u* → 0

$$\frac{Q_{L}}{Q_{F}}=\prod_{i=1}^{N-1}\frac{1+u(U_{L}^{i}-1)}{1+u(U_{F}^{i}-1)}\approx \prod_{i=1}^{N-1}[1+\left(U_{L}^{i}-U_{F}^{i}\right)u+\delta ]$$ (25)

$$\begin{array}{l} \frac{Q_{L}}{Q_{F}}\approx \prod_{i=1}^{N-1}[1+\left(U_{L}^{i}-U_{F}^{i}\right)u+\delta ]\\ \approx \prod_{i=1}^{N-1}[1+(\frac{i-1}{N-1}\left[P\left(C\right)\frac{(C_{1}-1)(1-e^{-rt_{2}})bR}{NrC_{1}}-C+\mu P\left(C\right)T\right]\\ +\frac{N-i}{N-1}\left[P\left(C\right)\frac{(C_{1}-1)(1-e^{-rt_{1}})aR}{NrC_{1}}+P\left(C\right)\frac{(C_{1}-1)(e^{-rt_{1}}-e^{-rt_{2}})bR}{NrC_{1}}-C+\mu P\left(C\right)T\right]\\ -\frac{i}{N-1}\left[P\left(C\right)\frac{(C_{1}-1)(e^{-rt_{1}}-e^{-rt_{2}})bR}{NrC_{1}}+\left(2-\mu \right)P\left(C\right)T\right]+\frac{N-i-1}{N-1}(-R^{*}))u+\delta ] \end{array}$$ (26)

Extract the common factor containing *T* or *μ*

$$\frac{Q_{L}}{Q_{F}}\approx \prod_{i=1}^{N-1}[1+\frac{N+i-1}{N-1}P\left(C\right)Tu\mu +\delta +\xi ]$$ (27)

All the previous conclusions still hold. And **Conclusion 3**. strategy “L” invades with the differential coefficient increases.

### Simulation

The model has been implemented in R studio(version 3.6.3) and cascading style sheets(CSS), All the R code, server code and dataset are all attached.

The relationship between evolutionary stability strategy and the strength of incentive *T* can be simulated according the Table 1(*N* = 100, *C*_1_ = 2, *a* = 0.4, *b* = 0.7, *R* = 1000, *C* = 100, *P*(*C*) = 0.4, *r* = 0.015, *t*_1_ = 100, *t*_2_ = 300, −R* = −100, mutation rate = 0.01).

**Fig 1:**
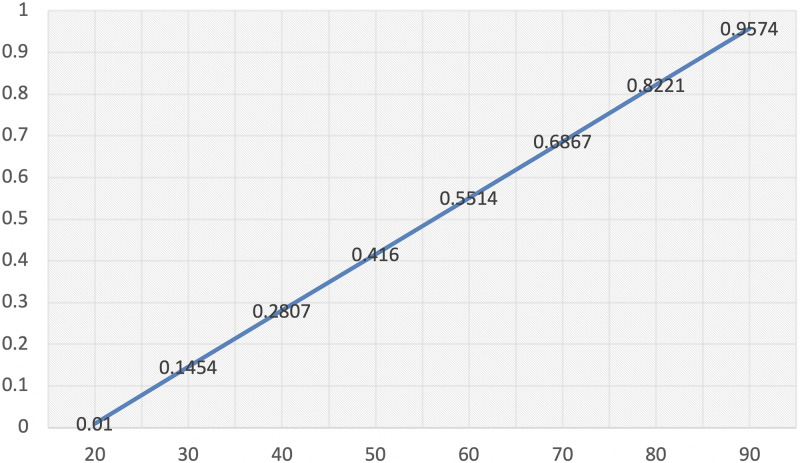
The X axis is the strength of incentive *T*, and the Y axis is the evolutionary stability strategy of $\frac{i}{N}$. dx.doi.org/10.17504/protocols.io.bgbfjsjn.

Fig 1 has shown that the dataset has a negative intercept, which means incentive has threshold effect, when the advantage of innovate is small enough, it may be offset by the randomness of the evolution process in finite population [68] as we discussed in the Chapter1.

**Conclusion 4**: Strength of incentive *T* has threshold effect.

Further, the relationship between evolutionary stability strategy and coefficient *μ* under different strength of incentive *T* can be simulated according the Table 2

**Fig 2:**
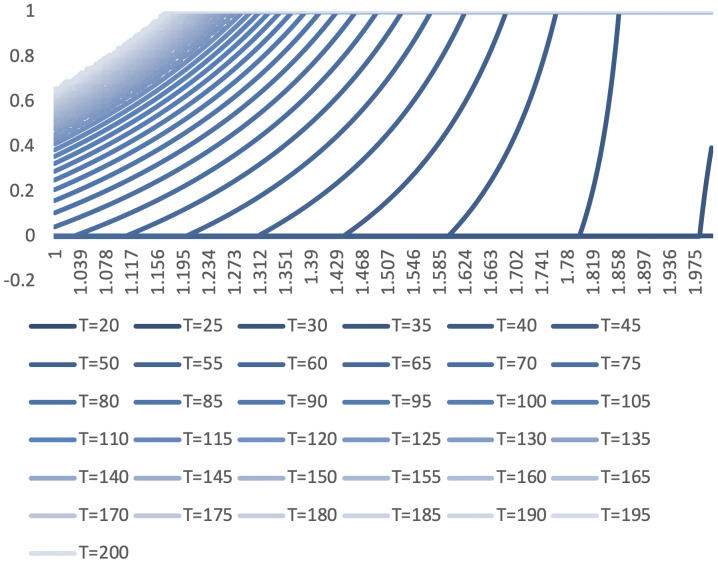
The X axis is the coefficient *μ* under different *T*, and the Y axis is the evolutionary stability strategy of $\frac{i}{N}$. dx.doi.org/10.17504/protocols.io.bgbgjsjw.

We take [*T* = 85, *μ* = 1.297, ESS = (0,0.6774,1)] in Fig 2 as an example, different *u* = 0.5/0.9 leads to different invasion dynamics and replacement probabilities(The strength of natural selection doesn’t change the ESS) in Table 3.

**Table 3:** Evolutionary game parameters with different *u*.

	*u* = 0.5	*u* = 0.9
innovators’ invasion dynamics	7.3887	13.2996
non-innovators’ invasion dynamics	-3.805	-6.849
innovators’ replacement probabilities	2.4313	2.8564
non-innovators’ replacement probabilities	0	0

Higher *u* leads to a higher replacement probabitity and a higher invasion dynamic compared with the lower one in Table 3, which confirmed **conclusion 1**.

Then we compare [*T* = 85, *μ* = 1.297, ESS = (0,0.6774,1), *u* = 0.5] and [*T* = 150, *μ* = 1.085, ESS = (0,0.6775,1), *u* = 0.5] which are both in Fig 2. Though different combinations have almost the same ESS, they still lead to different invasion dynamic and replacement probabilities in Table 4.

**Table 4:** Evolutionary game parameters with different μ*T*.

	*T* = 85, *μ* = 1.297, *u* = 0.5	*T* = 150, *μ* = 1.085, *u* = 0.5
leading innovators’ invasion dynamics	7.3887	17.7331
following innovators’ Invasion dynamics)	-3.805	-8.803
leading innovators’ Replacement Probabilities	2.4313	1.3064
following innovators’ Replacement Probabilities	0	0

Interestingly, [*T* = 85, *μ* = 1.297] has a higher replacement probability but a lower invasion dynamics for leading innovators compared with [*T* = 150, *μ* = 1.085] in Table 4.

**Conclusion 5**: With the same ESS, combination which has higher differential coefficient *μ* makes strategy “L” invades faster but more unstable than others.

## Results

With the equations derived in Chapter 3.1, we can summarize the dynamic nature of the relationship between benefits of different strategies and the probabilities of selecting them

$$\frac{Q_{V}}{Q_{D}}\approx \prod_{i=1}^{N-1}[1+\left(U_{V}^{i}-U_{D}^{i}\right)u+\delta ]\approx \prod_{i=1}^{N-1}\left[1+P\left(C\right)Tu+\delta +\eta \right]$$ (28)

$$\frac{Q_{L}}{Q_{F}}\approx \prod_{i=1}^{N-1}[1+\frac{N+i-1}{N-1}P\left(C\right)Tu\mu +\delta +\xi ]$$ (29)

Further more, the strength of natural selection, the strength of incentives and differential coefficient are related to variations in participants strategies over time.

In the first-order free riding problem, the strength of incentive *T* and strength of natural selection *u* both play a positive role in promoting strategy “V” to become evolutionary stable strategy. The simulation results are also consistent with the conclusions, more specifically, strategies with higher *T* or *u* has a higher invasion dynamic and replacement probability.

The previous conclusions still hold in the second-order free riding problem, we also find strategy “L” invades with the differential coefficient increases. So the strength of incentive *T*, strength of natural selection *u* and differential coefficient *μ* all play a positive role in promoting strategy “L” to become evolutionary stable strategy. The simulation results are still consistent with those, higher *T*, *u*, *μ* may have a higher invasion dynamic and replacement probability.

Strength of incentive *T* has threshold effect, too slight *T* makes no effect due to the randomness of the evolution process in finite size. Higher *T* often leads to better results, however, it is unwise to blindly increase *T* for it would be a waste of limited public resources. More micro observation perspectives and complex research methods are needed to deeper analysis.

We also find that different combinations of incentive methods *μT* may have the same evolutionary stable strategy. But the combination with higher *μ* has a higher invasion dynamic but lower replacement probability, which can make policy achieves the evolutionary stable strategy faster but more unstable. It is consistent with theory of public goods: collective incentive is not enough to strive for certain public goods, in contrast, though it deviates from the principle of fairness and even social stability, the reward gotten by an individual in an organization may become a selective incentive mechanism to make more contributions to the organization [69] [70].

## Discussion

This paper presented a two-stage stochastic evolutionary game model in finite population, each participant can choose strategy “V” or strategy “D”, strategy “L” or strategy “F” respectively in two stages. According to the birth-death algorithm of Moran process, we constructed the linear fitness equation of strategies to describe the expected payoff, and used the Markov probability transfer matrix of stochastic process to calculate the probability of strategy “V” and strategy “L” becoming the final result under different *T*, *u*, *μ* combinations. The simulation produces results which are consistent with the theoretical conclusions, moreover proved that there is threshold effect and optimal range in the selection of policy combinations.

In practical implications, we closed the gap of research on the analysis of green technology innovation by using stochastic evolutionary game, provided the key variables of green technology innovation incentive and principles for the environmental regulation policy making.

In a broader sense, this paper illustrates that economic society is a non-linear complex system, which means that policies aimed at promoting a particular aspect often produces unexpected results [71] [72], so it is very difficult to formulate policies reasonably and make them achieve the expected results [73]. Therefore, Professionalism and foresight are needed, any kind of policy should be carefully chosen.

Although this paper uses complicated interdisciplinary computing method in order to be as close to reality as possible, there are still some aspects that have not been mentioned, which would be our future research directions:

In reality, preference changes: participants can acquire new preferences or modify existing ones after learning the relationship between their strategy and rewarding [74]. A few assumptions of preference changes have been implicated in models of decision-making in psychology and behavioral economics [75] [76] [77] [78] [79] [80]. Besides, in large-scale group decision making methods, it is more realistic to assume that each participant has only a limited number of contacts which will be updated according to some rules, therefore participants would be divided into smaller subgroups and some of them may be clustered. Social network relations have been taken into consideration in some researches [81] [82] [83] [84] [85]. If we can simulate group decision-making process by using the fuzzy or variable preferences and social network model including hierarchy and aggregation structure [86], it will help us to understand the evolution process and promotion mechanism of behavior in more practical and intuitive aspects.

## Supporting information

S1 Data (ZIP)

## References

[1] Von Neumann J, Morgenstern O. Theroy of Game and Economic Behavior. Princenton:Princenton University Press 1944.

[2] Imhof L A. Evolutionary game dynamics under stochastic influences. Jahresbericht der deutschen Mathematiker-vereinigung. 2005;107(4): 197–213.

[3] Perc M. Phase transitions in models of human cooperation. Physics Letters A. 2016; 380(36): 2803–2808.

[4] Simon H A. Models of man: Social and rational American Catholic Sociological Review. 1957;8(3): 236.

[5] Smith J M. Evolution and the Theory of Games. Cambridge: Cambridge University Press 1982.

[6] Taylor P D, Jonker I B. Evolutionarily stable strategies and game dynamics. Levines Working Paper Archive. 1978;40(1–2): 145–156.

[7] Axelrod R, Hamilton W D. The evolution of cooperation. Science. 1981;211(4489): 13901396. 10.1126/science.7466396 7466396

[8] Hofbauer J Sigmund K. Evolutionary game dynamics. Bulletion of the American Mathematical Society. 2003;40(4): 479–519.

[9] Hofbauer J, Sigmund K. Evolutionary Games and Population Dynamics. Cambridge:Cambridge University Press 1998.

[10] Nowak M A, Sigmund K. Evolutionary dynamics of biological games. Science. 2004;303(5659): 793 10.1126/science.1093411 14764867

[11] Nowak MA. Evolutionary Dynamics: Exploring the Equations of Life. Cambridge:Harvard University Press 2006.

[12] Gintis H. Game Theory Evolving. Princeton: Princeton University Press 2000.

[13] Traulsen A Christoph H. Stochastic evolutionary game dynamics. Reviews of Nonlinear Dynamics and Complexity. 2009;2: 25–61.

[14] Cabrales A. Stochastic replicator dynamics. International Economic Review. 2000;41(2): 451481.

[15] Cressman R, Vickers G T. Spatial and density effects in evolutionary game theory. Journal of Theoretical Biology. 1997;184(4): 359 10.1006/jtbi.1996.0251 9082071

[16] Hutson V C L, Vickers G T. Travelling waves and dominance of ESS. Journal of Mathematical Biology.1992;30(5): 457–471.

[17] Brunnermeier, Cohen. Determinats of environmental innovation in US manufacturing industries. Journal of Environmental Economics and Management. 2003;45: 278–293.

[18] Zhao Rui, Zhou Xiao. For the sustainable performance of the carbon reduction labeling policies under an evolutionary game simulation. Technological Forecasting and Social Change. 2016;112(11): 262–274.

[19] Zglobisz N, Castillo Castillo A Grimes S Jones P. Influence of UK energy policy on the deployment of anaerobic digestion. Energy Policy. 2010;38(10): 5988–5999.

[20] Bohannan Chmristina, Hovenkamp Herbert. Creation Without Restraint: Promoting Liberty and Rivalry in Innovation. Oxford University Press, 2012, p,XI.

[21] Boulanger P M, Brechet T. Models for policy-making in sustainable development: the state of the art and perspectives for research. Ecol. Econ. 2005;55(3):337–350.

[22] Pelin D, Effie K. Stimulating different types of eco-innovation in the UK: Government policies and firm motivations. Ecological Economics.2011;70:1546–1557.

[23] Breschi S, Malerba F, Mancusi M L. Survival of innovative entrants in knowledge-based sectors In Malerba F., Knowledge intensive entrepreneurship and innovation systems: evidence from Europe. Abingdon: Routledge 2010 pp.136–153.

[24] Porter M E, Linde C V D. Toward a new conception of the environment-competitiveness relationship. Journal of Economic Perspectives.1995;9(4): 97–118.

[25] yagui Yu, Fangfang Zhang. The impact of government regulation on enterprise performance in environmental protection: an analysis based on Porter hypothesis. Ecological economy. 2016;1:99–101.

[26] Testa F, Iraldo F, Frey M. The effect of environmental regulation on firms’ competitive performance: The case of the building & construction sector in some EU regions. Environ Manag. 2011;92:2136–2144. 10.1016/j.jenvman.2011.03.039 21524840

[27] Rassier D G, Earnhart D. Effects of environmental regulation on actual and expected profitability. Ecol Econ.2015;112: 129–140.

[28] Ming Zhang, Hao Li. New evolutionary game model of the regional governance of haze pollution in China. Applied Mathematical Modelling. 2018;63: 577–590.

[29] Zhang Suyong, Wang Chuanxu, Yu Chao. The evolutionary game analysis and simulation with system dynamics of manufacturer's emissions abatement behavior under cap-and-trade regulation. Applied Mathematics and Computation. 2019;355(15): 343–355.

[30] Yuan Liang, He Weijun. Transboundary water sharing problem: a theoretical analysis using evolutionary game and system dynamics. Journal of Hydrology. 2020;582(3): 124521.

[31] Malakpour Estalaki Siamak. Hybrid Agent-Based framework for simultaneous water allocation and cropping pattern optimization. Environmental Earth Sciences. 2015;73(8): 4201–4213.

[32] Huang J, Leng M M, Liang L P. Promoting electric automobiles: Supply chain analysis under a government’s subsidy incentive scheme. IIE Transactions.2013;45(8): 826–844.

[33] Zhiguo Wang, Lei Li, Shanlin Yang, et al Research on government low carbon regulation to guide enterprise low carbon technology innovation under dynamic game. China management science. 2016;12: 139–147.

[34] Krass D, Nedorezov T, Ovchinnikov A. Environmental taxes and the choice of green technology. Production and Operations Management. 2013;22(5): 1035–1055.

[35] Jianzhong Xu, Jun Guan, Xiaoya Zhu. Evolutionary game research on the influence of government behavior on the choice of green innovation mode of manufacturing enterprises. Operations research and management. 2017;9: 68–77.

[36] Aiyun Nie, Xiaogang he. Green technology innovation of enterprises: environmental regulation and policy combination. Reform.2012;4: 102–108.

[37] Claussen J C, Traulsen A. Non-gaussian fluctuations arising from finite populations: Exactresults for the evolutionary Moran process. Physical Review.2005;71(2): 025101. 15783363 10.1103/PhysRevE.71.025101

[38] Traulsen A, Claussen J C. Similarity-based cooperation and spatial segregation. Physical Review.2004;70(4): 46128. 10.1103/PhysRevE.70.046128 15600481

[39] Taylor C, Iwasa Y, Nowak M A. A symmetry of fixation times in evolutionary dynamics. Journal of Theoretical Biology.2006;243(2): 245–251. 10.1016/j.jtbi.2006.06.016 16890959 PMC2879639

[40] Ohtsuki H, Hauert C, Lieberman E. A simple rule for the evolution of cooperation ongraphs and social networks. Nature. 2006;441(7092): 502–505. 10.1038/nature04605 16724065 PMC2430087

[41] Brauchli K, Killingback T, Doebeli M. Evolution of cooperation in spatially structured popula-tions. Journal of Theoretical Biology.1999;200(4): 405–417. 10.1006/jtbi.1999.1000 10525399

[42] Fudenberg D, Nowak M A, Taylor C, et al Evolutionary game dynamics in finite populationith strong selection and weak mutation. Theoretical Population Biology. 2006;70(3): 352–363. 10.1016/j.tpb.2006.07.006 16987535 PMC3279757

[43] Antal T, Scheuring I. Fixation of strategies for an evolutionary game in finite populations. Bulletin of Mathematical Biology.2006;68(8): 1923–1944. 10.1007/s11538-006-9061-4 17086490

[44] Křivan Vlastimil. Beyond replicator dynamics: From frequency to density dependent models of evolutionary games. Journal of Theoretical Biology.2018;455(10): 232–248. 29990466 10.1016/j.jtbi.2018.07.003

[45] Fudenberg D, Nowak M A, Taylor C, et al Evolutionary game dynamics in finite populations with strong selection and weak mutation. Theoretical Population Biology. 2006;70(3): 352–363. 10.1016/j.tpb.2006.07.006 16987535 PMC3279757

[46] Liu X, Pan Q, Kang Y, He M. Fixation probabilities in evolutionary games with the Moran and Fermi processes. J. Theor. Biol. 2015;364:242–248. 10.1016/j.jtbi.2014.08.047 25218430

[47] Moran P A P. The statistical process of evolutionary theory. Clarendon Press, Oxford 1962.

[48] Imhof L A, Nowak M A. Evolutionary game dynamics in a Wright-Fisher process. Journal of Mathematical Biology. 2006;52(5): 667–681. 10.1007/s00285-005-0369-8 16463183 PMC3279756

[49] Tataru P, Bataillon T, Hobolth A. Inference under a Wright-Fisher model using an accurate beta approximation. Genetics. 2015;201(3): 1133 10.1534/genetics.115.179606 26311474 PMC4649640

[50] Liu X, Pan Q, Kang Y, He M. Fixation probabilities in evolutionary games with the Moran and Fermi processes. J. Theor. Biol. 2015;364:242–248. 10.1016/j.jtbi.2014.08.047 25218430

[51] Whigham P A, Dick G. Evolutionary dynamics for the spatial Moran process. Kluwer Academic Publishers 2008.

[52] Taylor C, Fudenberg D, Sasaki A, et al Evolutionary game dynamics in finite populations. J. Bulletin of mathematical biology. 2004;66(6): 1621–1644. 10.1016/j.bulm.2004.03.004 15522348

[53] Nowak M A, Sasaki A, Taylor C, et al Emergence of cooperation and evolutionary stability in finite populations. Nature. 2004;428 (6983): 646–650. 10.1038/nature02414 15071593

[54] Traulsen A, Shoresh N, Nowak M A. Analytical results for individual and group selection of any intensity. Bulletin of mathematical biology.2008;70(5): 1410–1424. 10.1007/s11538-008-9305-6 18386099 PMC2574888

[55] Traulsen A, Hauert C. Stochastic evolutionary game dynamics. Wiley-VCH 2009.

[56] caichun Chai, Tiaojun Xiao, Tiantian Xu. The evolution of manufacturer's production strategy based on Moran process. System engineering theory and practice. 2015;9:2262–2270.

[57] Xianjia Wang, Qilong He, Ji Quan, et al Evolution of consumer crowdfunding strategy based on Moran process. Operations research and management.2017;11: 105–110.

[58] Laird Robert A. Sequential interactions-in which one player plays first and another responds—promote cooperation in evolutionary-dynamical simulations of single-shot Prisoner's Dilemma and Snow drift games. Journal of Theoretical Biology.2018;452(7):69–80. 29763610 10.1016/j.jtbi.2018.05.007

[59] Tongliang An, Shaodong Zhou, Jiancai Pi. Incentive Effect of R&D Subsidies on Independent Innovation of Chinese Enterprises. Economic Research. 2009;44 (10):87–98.

[60] Fudenberg D, Nowak M A, Taylor C, et al Evolutionary game dynamics in finite populations with strong selection and weak mutation. Theoretical population biology,2006;70(3): 352–363. 10.1016/j.tpb.2006.07.006 16987535 PMC3279757

[61] Traulsen A, Hauert C. Stochastic evolutionary game dynamics. Wiley-VCH 2009.

[62] Yefeng Chen, Hang Ye, Dingding wang. The Theory of Social Preference Beyond Economic People: A Review Based on Experimental Economics. Nankai Economic Research.2012;1: 63–100.

[63] Nowak M A, Sigmund K. Evolutionary dynamics of biological games. Science.2004;303(5659): 793 10.1126/science.1093411 14764867

[64] caichun Chai, Tiaojun Xiao, Tiantian Xu. The evolution of manufacturer's production strategy based on Moran process. System engineering theory and practice. 2015;9:2262–2270.

[65] Nowak M. Evolutionary Dynamics: Exploring the Equations of Life. Cambridge: Harvard University Press 2006.

[66] Ostrom Elinor, Governing the Commons: The Evolution of Institutions for Collective Action. Cambridge: Cambridge University Press 1990.

[67] Marwell Gerald, Oliver Pamela & Prahl Ralph, Social Networks and Collective Action: A Theory of Critical Mass, III. American Journal of Sociology.1994.

[68] hang Ye. Social dilemma and social justice in public cooperation—interdisciplinary study of economics based on computer simulation. Economic research. 2012;47(8): 132–145.

[69] Ostrom E. Reformulating the Commons. Swiss Political Science Review.2000;6(1):29–52.

[70] Tortajada C. Water Governance: Some Critical Issues. International Journal of Water Resources Development. 2010;26(2):297–307.

[71] Petrović Marko. Should I stay or should I go? An agent-based setup for a trading and monetary union. Journal of Economic Dynamics and Control.2020;113:103866.

[72] Luis R, Izquierdo. An introduction to ABED: Agent-based simulation of evolutionary game dynamics. Games and Economic Behavior.2019;118: 434–462.

[73] Hefeng Tong, Yang Yang, Jingyi Wang, et al China's green economy development prospect: scenario analysis based on system dynamics model. China Soft Science. 2015;6: 20.

[74] Sutton R S, Barto A G. Reinforcement Learning: An Introduction. MIT Press 1998.

[75] Cushman F. Rationalization is rational. Behavioral and Brain Sciences. 2019;1:69. 10.1017/S0140525X19001730 31133084

[76] Hornsby A N, Evans T, Riefer P S, Prior R, Love B C. Conceptual Organization is Revealed by Consumer Activity Patterns. Computational Brain and Behavior.2019. 10.1007/s42113-019-00064-9 PMC7235073 32455337

[77] Schulz E, Bhui R, Love B C, Brier B, Todd M T, Gershman S J. Structured, uncertainty-driven exploration in real-world consumer choice. Proceedings of the National Academy of Sciences.2019;21:28. 10.1073/pnas.1821028116 PMC6628813 31235598

[78] Nilsson A, Bergquist M, Schultz W P. Spillover effects in environmental behaviors, across time and context: a review and research agenda. Environ. Educ. Res. 2017;23 (4):573–589.

[79] Moutoussis M, Dolan R J, Dayan P, How People Use Social Information to Find out What to Want in the Paradigmatic Case of Inter-temporal Preferences. PLoS Comput. Biol. 2016;1:17. 10.1371/journal.pcbi.1004965 PMC4957786 27447491

[80] Calluso C, Tosoni A, Pezzulo G, Spadone S, Committeri G. Interindividual variability in functional connectivity as long-term correlate of temporal discounting. PLoS One.2015;10. 10.1371/journal.pone.0119710 PMC4361316 25774886

[81] Chu Junfeng, Wang Yingming, Liu Xinwang, Liu Yicong. Social network community analysis based large-scale group decision making approach with incomplete fuzzy preference relations, Information Fusion. 2020.

[82] Wasserman S, Faust K. Social network analysis: Methods and applications. Cambridge university press 1994.

[83] Ding R X, Palomares I, Wang X, Yang G R, Liu B, Dong Y. Large-Scale Decision-Making: characterization, taxonomy, challenges and future directions from an Artificial Intelligence and applications perspective. Information Fusion. 2020.

[84] Huang M, Zou G, Zhang B, Liu Y, Gu Y, Jiang K. Overlapping community detection in heterogeneous social networks via the user model. Information Sciences.2018;432:164–184.

[85] Tian Z P, Nie R X, Wang J Q. Social network analysis-based consensus-supporting framework for large-scale group decision-making with incomplete interval type-2 fuzzy information, Information Sciences. 2019;502: 446–471.

[86] G Iacobelli, D Madeo. Lumping evolutionary game dynamics on networks. Journal of Theoretical Biology. 2016;407(10): 328–338. 27475842 10.1016/j.jtbi.2016.07.037

